# Breast Cancer in Ghana: Demonstrating the Need for Population-Based Cancer Registries in Low- and Middle-Income Countries

**DOI:** 10.1200/JGO.2016.006098

**Published:** 2017-03-27

**Authors:** Abigail S. Thomas, Kelley M. Kidwell, Joseph K. Oppong, Ernest K. Adjei, Ernest Osei-Bonsu, Angela Boahene, Evelyn Jiagge, Kofi Gyan, Sofia D. Merajver

**Affiliations:** **Abigail S. Thomas**, **Kelley M. Kidwell**, and **Sofia D. Merajver**, University of Michigan School of Public Health, and **Evelyn Jiagge**, University of Michigan School of Medicine Ann Arbor, MI; **Joseph K. Oppong**, **Ernest K. Adjei**, **Ernest Osei-Bonsu**, and **Angela Boahene**, Komfo Anokye Teaching Hospital, Kumasi, Ghana; and **Kofi Gyan**, Henry Ford Hospital, Detroit, MI

## Abstract

**Purpose:**

Breast cancer, the most common cancer worldwide, is the leading cause of cancer mortality in Ghanaian women. Previous studies find Ghanaian women are diagnosed at a younger age and at more advanced stages (III and IV), and have tumors with characteristics similar to African American women. We sought to remedy gaps in knowledge about breast cancer survival in Ghana and its relation to demographic and biologic factors of the tumors at diagnosis to assist in cancer control and registration planning.

**Methods:**

Individuals with a breast cancer diagnosis who sought care at Komfo Anokye Teaching Hospital from 2009 to 2014 were identified via medical records. Follow-up telephone interviews were held to assess survival. Kaplan-Meier plots and Cox proportional hazards models assessed survival associated with clinical and demographic characteristics.

**Results:**

A total of 223 patients completed follow-up and were analyzed. The median survival was 3.8 years. Approximately 50% of patients were diagnosed with grade 3 tumors, which significantly increased the risk of recurrence or death (hazard ratio [HR] for grade 2 versus 1, 2.98; 95% CI, 1.26 to 7.02; HR grade 3 *v* 1, 2.56; 95% CI, 1.08 to 6.07; *P* = .04). No other variables were significantly associated with survival.

**Conclusion:**

Higher tumor grade was significantly associated with shorter survival, indicating impact of aggressive biology at diagnosis on higher risk of cancer spread and recurrence. Contrary to prevailing notions, telephone numbers were not reliable for follow-up. Collecting additional contact information will likely contribute to improvements in patient care and tracking. A region-wide population-based active registry is important to implement cancer control programs and improve survival in sub-Saharan Africa.

## INTRODUCTION

Breast cancer is the most common cancer affecting women worldwide, with approximately 1.4 million patients diagnosed annually. Half of all patients with breast cancer occur in low- and middle-income countries (LMICs), which are experiencing a double burden of rising noncommunicable diseases with existing prevalent infectious diseases.^1,2^ The incidence-to-mortality ratio reveals much higher mortality in LMICs from all cancers, with 58% of breast cancer deaths occurring in these countries.^2,3^ This cancer burden is reflected in Ghana, a lower-middle–income country, because breast cancer is the most common cause of cancer deaths in Ghanaian women.^4^ Because Ghana has no population-based cancer registry, the best-known breast cancer incidences must be estimated from single-institution databases and range from 15.2 to 35 patients per 100,000 population.^2,5-8^

Although the incidence is currently low, it is expected to increase as Ghana’s population ages and a Western lifestyle is adopted.^9-11^ The lack of a population-based cancer registry is not unique to Ghana among African nations. The International Agency for Research on Cancer lists only four sub-Saharan population-based cancer registries: Kampala, Uganda; Harare, Zimbabwe; Blantyre, Malawi; and the Program on Mycotoxins and Experimental Carcinogenesis in South Africa; these represent < 5% of the sub-Saharan population.^7,12^

Therefore, all studies of breast cancer in Ghana use hospital-based data. These studies have found many differences in clinical course and markers in tumors from women diagnosed with breast cancer in Ghana compared with those in the United States. Previous studies indicate that Ghanaian women tend to present at a younger age, a more advanced stage (III and IV), have larger tumors, and have fewer tumors that are hormone positive.^4,13^ Primarily because of a younger age pyramid, African women tend to be diagnosed with breast cancer a decade younger than American women.^14^ The advanced stage at diagnosis indicates there are likely significant delays or deficiencies in multiple facets of the diagnostic process, but patients’ delay in seeking care has been reported to be 8 to 10 months after the onset of symptoms.^13,15^ In fact, most women tend to be unaware of the disease until there is a large palpable mass, and then they seek care.^16^ This delay could be attributed to stigma associated with breast cancer or difficulties accessing medical centers with cancer care, and usually results in the disease having progressed beyond an early stage when diagnosed, which is commonly associated with poorer prognoses.^7^ There are no current national breast cancer screening protocols in Ghana or countrywide literacy initiatives and, with limited availability of mammography or ultrasound machines, further health-system–centered delays in diagnosis ensue, even when patients seek care.^4^

Little is known about the survival of patients with breast cancer in Ghana. A study in 2001 found the 5-year survival rate to be 25.3%.^17^ At the time, this low survival rate could be attributed to low resources to access care, varying health behaviors and habits, health-system deficiencies, and paucity of knowledge or concern about breast cancer.^3,17-22^ This is a stark contrast to the estimated > 80% 5-year survival in high-resource areas, such as North America and Japan.^23^ Extrapolating from Western countries’ data, claims have been made that to decrease breast cancer mortality, both early detection and proper treatments must be integrated in LMICs.^3^ In Ghana, there is a paucity of critical data on breast cancer survival. This is crucial missing information that would help determine the factors that affect poor prognosis and survival, and also would provide a baseline for tracking the effectiveness of potential interventions aimed at ameliorating low survival. In previous work, we demonstrated challenges in navigation and completion of cancer therapies.^5^ Changes are being implemented in Ghana, in general, and in Kumasi, in particular, to address these deficits. However, the relationship between survival and biologic and demographic factors remains largely unknown.

Biologic factors affecting a tumor’s metastatic potential can also influence mortality. In this regard, poor prognosis from breast cancer in Ghanaians could partially be due to tumors having low rates of hormone-receptor positivity, because estrogen-receptor (ER) –positive and progesterone-receptor (PR) –positive breast cancers are associated with higher survival rates than triple-negative breast cancer (TNBC). TNBC includes those breast cancers that test negative for estrogen, progesterone, and human epidermal growth factor receptor 2 (HER2). Previous studies found that Ghanaians have a three-fold increase in TNBC rates compared with African Americans and white Americans. Specifically, in the study’s sample, 82% of Ghanaians were diagnosed with TNBC versus 26% of African Americans and 16% of white Americans.^18,24,25^ TNBC is typically diagnosed in premenopausal women and has been correlated with poorer prognosis.^26-29^ A previous study at Korle Bu Teaching Hospital found 49.4% of the tumors tested were hormone-receptor negative, with TNBC being the most common subtype.^4,18^

In our study, we sought to measure survival rates for breast cancer in Ghana and correlate it with demographic and biologic factors. Komfo Anokye Teaching Hospital (KATH) in Kumasi was chosen for this retrospective hospital-based breast cancer study because it is a large referral hospital for the Ashanti region, as well as the second largest teaching hospital in Ghana, and is located in the southern half of the country.^4,5,30^ The primary objectives of this study were to determine the age distribution and average survival time of patients with breast cancer who were diagnosed from 2009 to 2014 via a retrospective medical chart review and patient interviews. We also sought to explore which tumor characteristics (ie, grade and hormonal status) were correlated with survival.

## METHODS

### Study Population

The University of Michigan Institutional Review Board; the Committee on Human Research, Publications, and Ethics at Kwame Nkrumah University of Science and Technology School of Medical Sciences; and the KATH Research Committee approved the retrospective study. Patients who were newly diagnosed with breast cancer and sought care at KATH from 2009 to 2014 were identified using medical records written in English and archived in the breast care center, pathology, and radiation oncology departments. Data of patients with pathologically confirmed breast cancer were collected. Patients without pathologically confirmed breast cancer and those diagnosed with a nonmalignant breast disease (eg, fibroadenoma, gynecomastia, fat necrosis, or duct ectasia) were excluded from the data set. The data were linked from each department on date of birth and name because there is no common hospital identifier used for all departments.

### Data Variables

Demographic information, family history, contraception use, and breast cancer clinical pathologic characteristics (ie, date of diagnosis, grade of tumor, menstruation status at diagnosis, and hormone-receptor status) were extracted from the medical records. Age at diagnosis was registered in years. Family history, contraception use, and menstruation status at diagnosis were collected as a dichotomous variable (ie, yes/no), with the family history expanded to the relationship between the patient and the family member with cancer history. The length of contraception use was recorded in months. The date of diagnosis was the date a pathologist confirmed the biopsy specimen as malignant breast carcinoma. The grade of the tumor was identified in the pathology report from medical records, ranging from 1 to 3. At KATH, two pathologists consistently evaluate all breast biopsy specimens. ER or PR status was considered negative if < 10% of the cancer cells on the slide were positive. A score of 0 or 1+ on the HER2 receptor was considered negative. A TNBC was defined if the patient had negative ER, PR, and HER2 receptor status. The variables were considered missing if no staining was done.

### Follow-Up

If present, the contact telephone numbers in medical records were recorded for the follow-up interview. A database in the radiation oncology department was consulted to collect additional telephone numbers for patients who received treatment from 2009 to 2014.

After the collection of contact telephone numbers, an experienced oncology nurse conducted follow-up telephone interviews in Twi, the most common local language, to determine if the patient was alive and record recurrence events. Each follow-up interview lasted approximately 5 to 10 minutes and included collecting data on variables such as date of birth, date of diagnosis, family history of breast cancer, contraceptive use and length of use, menstruation status at diagnosis, and cancer recurrence events. Verbal informed consent was obtained before the interview. If the patient was deceased and a family member or friend answered the call, the nurse consented to that person and asked about the health of the patient before death. Participants in the telephone interviews were compensated with 10 Ghanaian cedi in telephone credit.

### Statistical Analysis

The data were analyzed using SAS version 9.4 (SAS Institute, Cary, NC). Descriptive statistics were conducted on the demographic variables. A Kaplan-Meier curve was plotted to assess disease-free survival. Survival time was calculated as the difference between date of diagnosis and date of death, recurrence, or last follow-up. A log-rank test was performed to compare the survival for patients diagnosed with TNBC versus all other hormone-status subtypes and to compare patients diagnosed with a tumor grades 1, 2, and 3. Fisher’s exact test was performed to assess the association between TNBC status and grade. Cox proportional hazards models assessed the size and significance of demographic and clinical variables on survival.

## RESULTS

There were 1,469 breast cancer patients identified via medical records during 2009 to 2014. Of these, 663 patients had useable telephone information. Thirty-five percent of telephoned numbers were answered (n = 229) and of those 97.4% (n = 223) resulted in evaluable interviews.

**Fig 1:**
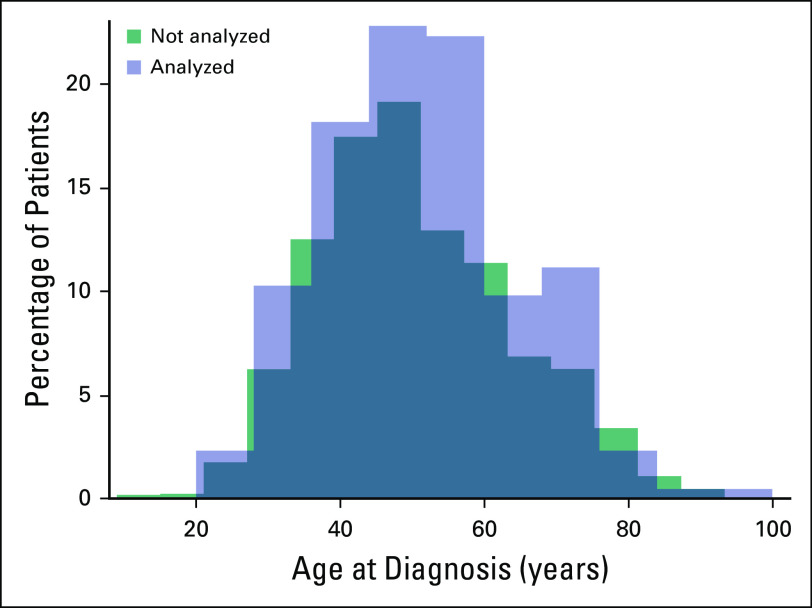
The distribution of age at diagnosis for the 223 patients included in this analysis from 2009 to 2014 and 1,246 patients not used in analysis. The average age at diagnosis was 51 years (standard deviation [SD], 14 years) and the median age was 50 years for those analyzed for survival. The average age at diagnosis for those not used in analysis was 50 years (SD, 14) and the median age was 48 years. A *t* test to determine significant difference in the distribution of age was performed (*P* = .22).

The demographic characteristics of the patients diagnosed between 2009 and 2014 are shown in Table 1. Nearly half (49.5%) of the patients whose data were used in analysis were diagnosed at grade 3, and only 12.4% of patients were diagnosed at grade 1. TNBC was diagnosed in 23.7% of patients. Male breast cancer accounted for six patients (2.7%) analyzed, which was considered a too-small sample size for a separate analysis. The average age at diagnosis was 51 years (standard deviation [SD], 14 years), ranging from 23 to 99 years for the 223 patients analyzed (Fig 1).

**Table 1:**
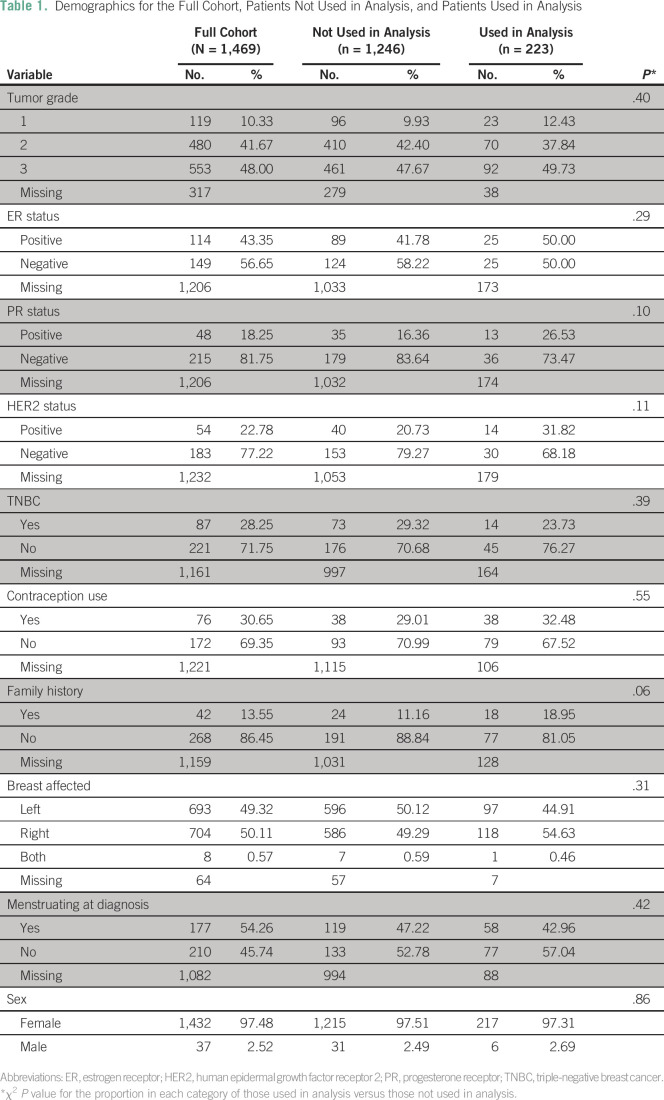
Demographics for the Full Cohort, Patients Not Used in Analysis, and Patients Used in Analysis

**Fig 2:**
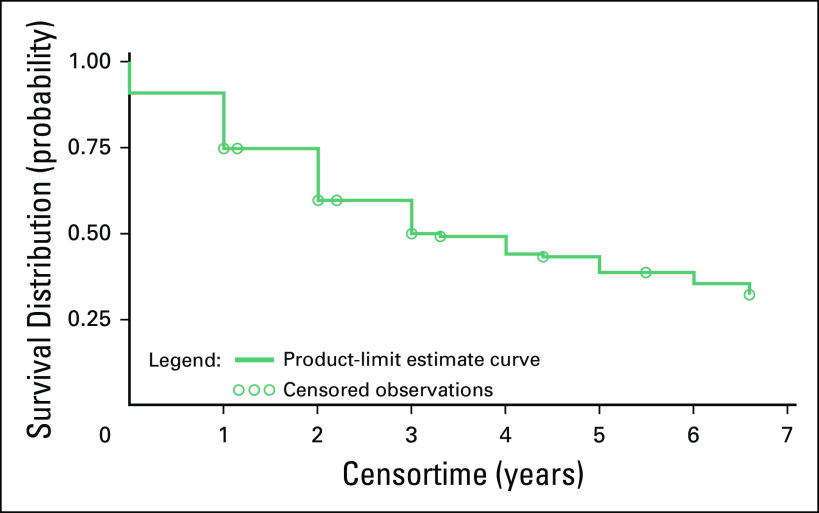
The Kaplan-Meier survival curve for all patients analyzed at Komfo Anokye Teaching Hospital from 2009 to 2014. The median time to death or recurrence was 3.8 years. The average time to follow-up was 2.4 years from the date of diagnosis.

**Fig 3:**
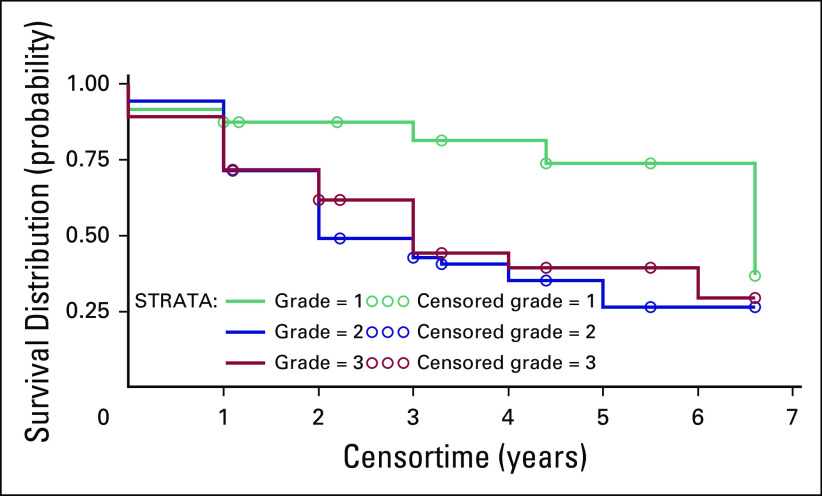
The survival curve by grade at diagnosis with tumor grades 1, 2, and 3 (*P* = .02) for the population at Komfo Anokye Teaching Hospital analyzed from 2009 to 2014.

The Kaplan-Meier survival curve is represented in Figure 2. The median time to death or recurrence was 3.8 years. There were 109 patients (48.9%) who had had a recurrence or died. Grade at diagnosis was significantly associated with survival. A higher grade was associated with increased risk of recurrence or death (hazard ratio [HR] for grade 2 *v* 1, 2.98; 95% CI, 1.26 to 7.02; HR for grade 3 *v* 1, 2.56; 95% CI, 1.08 to 6.07; *P* = .04). The log-rank test to observe the difference of survival among patients diagnosed at grades 1, 2, and 3 was similarly significant (*P* = .02); the survival curve and the number of those at risk are shown in Figure 3.

**Fig 4:**
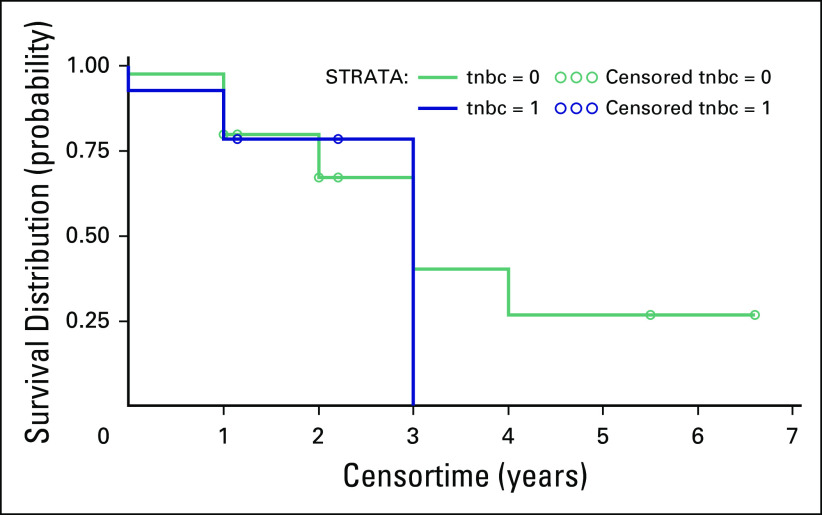
The survival curve for triple-negative breast cancer (TNBC) patients compared with all other hormone statuses (*P* = .82) for the population analyzed at Komfo Anokye Teaching Hospital from 2009 to 2014. Those with TNBC hormone status of 1 were positive for TNBC and those with TNBC hormone status of 0 were all other hormone-status patients.

The log-rank test to compare survival between patients with TNBC versus those of all other hormone-status subtypes was not statistically significant (*P* = .82); the survival curve and those at risk are shown in Figure 4. The mean age of patients with TNBC was 53 years (SD, 12 years), ranging from 35 to 77 years. The mean age of patients with any other hormone-receptor status also was 53 years (SD, 14 years), ranging from 28 to 82 years. The distribution of ages at diagnosis did not show significant differences by TNBC status (*P* = 1.00). The patient’s grade by TNBC status was also assessed (Table 2) and there was no statistically significant association between grade and TNBC status (*P* = .55).

**Table 2:**
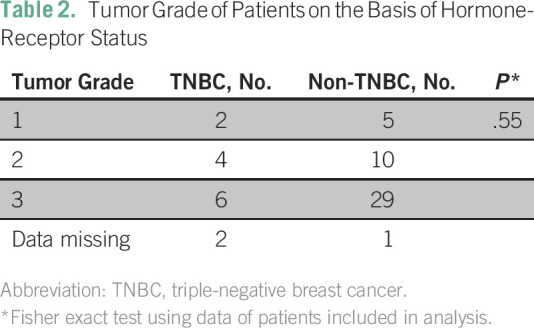
Tumor Grade of Patients on the Basis of Hormone-Receptor Status

## DISCUSSION

To implement effective cancer control programs that allow patients easy navigation of treatment and follow-up, it is important to understand survival in health care settings where diagnosis and treatment can be carried out. In the absence of a population-based registry, a logical starting point in understanding survival is at the most advanced cancer centers. The median survival time from diagnosis to either death or recurrence at KATH was 3.8 years. This is the first measurement of overall and disease-free survival of breast cancer at the most advanced tertiary cancer care center in the Ashanti region, serving the population of the entire northern sector of Ghana.^31^

Patients were diagnosed a decade younger when compared with white Americans; this was not surprising, because the median female age in Ghana is 21 years compared with 39 years in the United States.^14,32^ The average age at diagnosis of 51 years reported here is slightly older than the 49.2 years reported previously.^4^

There was a significant difference in time to death by tumor grade, demonstrating in this African cohort the likely impact of aggressive biology at diagnosis on higher risk of early cancer spread and recurrence. In contrast, the survival of patients with TNBC status versus all other subtypes was not statistically significant, unlike in the United States, but this may be due to missing and sparse data, and may not be representative of the KATH catchment area.

KATH’s catchment area includes those living in the Ashanti, Brong Ahafo, Central, Western, Eastern, Northern, Upper East, Upper West, and Volta regions of Ghana. According to the 2010 census, this area includes approximately 14 million people.^31^ In this region, there are several active centers where patients with breast cancer are diagnosed. With KATH’s catchment area and breast cancer incidence ranging from 15.2 to 35 patients per 100,000 population per year, it can be estimated that there would be 9,000 to 23,000 patients in the catchment area during a 5-year period, should the region have an active cancer registry. Our study found that 1,469 patients were reported at KATH from 2009 to 2014, accounting for 6% to 16% of the estimated breast cancer occurrences. Although other regional and private hospitals are known to also evaluate patients with breast cancer, KATH is considered the hospital where most patients are ascertained, clearly implying that a large percentage of breast cancers in the Kumasi area are likely to remain undiagnosed, indicating a pressing need to increase literacy about the importance of breast cancer diagnosis and prompt initiation of effective therapies.

There were fewer patients diagnosed at late stage (ie, III/IV) breast cancer: 49% compared with previous studies that reported 60% of Ghanaians in the Korle Bu Teaching Hospital area.^5,13^ This could be due to a modest overall improvement in breast cancer treatment or to regional differences in breast cancer care. Previous studies estimate the 5-year survival varied by stage at diagnosis and country, ranging from 40% to 80% with better survival correlating with higher-income countries.^23^ To our knowledge, this is the first study of breast cancer survival focused on a major teaching hospital in Ghana, which currently is the best source of records available. It is somewhat surprising that almost 50% of the patients analyzed were diagnosed at a late stage (ie, III/IV) but still the 5-year survival, at 39%, was higher than expected for such aggressive tumors. This rate is also higher compared with an earlier study that estimated Ghana’s 5-year survival rate for breast cancer to be approximately 25.3%, possibly showing an improvement in breast cancer care in the past 8 to 10 years.^17^ During this time, collaborations between academic institutions in the United States, other countries, and US health agencies have carried out diverse projects to improve cancer control. Although the possible improvement in breast cancer survival cannot be proven to be due to these efforts, it is clear they have led to improvements in the hospital base registry of new breast cancer patients.

When comparing the survival of patients with TNBC with that of other patients of other hormone status, there was not a significant difference in survival, unlike other studies that found TNBC status was associated with poorer survival.^3^ This discrepancy is most likely due to the insufficient sample size of patients with complete hormone-status information in the study. This is an area of cancer care that needs major improvement, because receptor status evaluation in all tumors is universally considered essential for appropriate breast cancer management. In Ghana, receptor evaluation can often be an out-of-pocket expense, possibly not affordable by many patients who forgo or postpone the evaluation.

Although there were fewer patients identified than would have been estimated, given the importance of KATH as a regional treatment center for cancer care, even fewer patients were successfully contacted for a follow-up telephone interview. In seeking to contact the patients by what was assumed to be the most effective manner, it was found that the nurse could only contact 35% of those who had telephone numbers listed in the medical record. Discontinued service and telephone numbers assumed by unrelated persons were the main reasons for failure to establish contact. It is possible that the survival measurement was affected by the limitations of telephone contacts, which could be further limited by the socioeconomic status of those who did provide telephone numbers. It is also possible that those with the financial means to have a mobile telephone would also have the means to seek treatment earlier, possibly presenting with smaller tumors at earlier stages. Therefore, we conclude that accessing patients via telephone only was not reliable for as large a percentage of the cohort as was anticipated by the hospital personnel. Thus, our study results strongly suggest that the follow-up of patients may improve if additional telephone numbers or other contact information were collected and used regularly, because this was a major challenge in the follow-up interviews. Once contacted, nearly all patients were willing to provide information about their health. An electronic database registry with active real-time updates would also improve follow-up care and understanding of whether treatments are completed and their impact on survival. Ideally, a population-based cancer registry would be created to accurately find the incidence and prevalence of any type of cancer in Ghana. This would yield a firm understanding of the burden of disease and the effectiveness of treatments for different cancer types.

This study supports the need for more research to ascertain if and where in the Ashanti region other patients with breast cancer were diagnosed and how they were treated and followed. In addition to providing better estimates of the incidence of breast cancer, such research would also help institute a real-time electronic database to gather detailed data on molecular markers, treatment, and survival data for all patients with cancer.
